# The Joint Effect of Physical Multimorbidity and Mental Health Conditions Among Adults in Australia

**DOI:** 10.5888/pcd17.200155

**Published:** 2020-12-10

**Authors:** Marie Ishida, Emily SG Hulse, Robert K Mahar, Jane Gunn, Rifat Atun, Barbara McPake, Naveen Tenneti, Kanya Anindya, Gregory Armstrong, Patrick Mulcahy, Will Carman, John Tayu Lee

**Affiliations:** 1 Nossal Institute for Global Health, Melbourne School of Population and Global Health, University of Melbourne, Melbourne, Australia; 2 Centre for Health Policy, Melbourne School of Population and Global Health, University of Melbourne, Melbourne, Australia; 3 Centre for Epidemiology and Biostatistics, Melbourne School of Population and Global Health, University of Melbourne, Melbourne, Australia; 4 Victorian Comprehensive Cancer Centre, University of Melbourne, Melbourne, Australia; 5 Department of General Practice, Melbourne Medical School, University of Melbourne, Melbourne, Australia; 6 Department of Global Health and Social Medicine, Harvard Medical School, Harvard University, Boston, Massachusetts; 7 Department of Global Health and Population, Harvard TH Chan School of Public Health, Harvard University, Boston, Massachusetts; 8 Department of Primary Care and Public Health, School of Public Health, Imperial College London, United Kingdom

## Abstract

**Introduction:**

The prevalence of chronic physical and mental health conditions is rising globally. Little evidence exists on the joint effect of physical and mental health conditions on health care use, work productivity, and health-related quality of life in Australia.

**Methods:**

We analyzed data from the Household, Income and Labour Dynamics in Australia (HILDA) Survey, waves 9 (2009), 13 (2013), and 17 (2017). Economic effects associated with multimorbidity were measured through health service use, work productivity loss, and health-related quality of life. We used generalized estimating equations to assess the effect of the association between physical multimorbidity and mental health conditions and economic outcomes.

**Results:**

From 2009 through 2017 the prevalence of physical multimorbidity increased from 15.1% to 16.2%, and the prevalence of mental health conditions increased from 11.2% to 17.3%. The number of physical health conditions was associated with the number of health services used (general practitioner visits, incidence rate ratio = 1.41), work productivity loss (labor force participation, adjusted odds ratio = 0.71), and reduced health-related quality of life (SF-6D score: Coefficient = −0.03). These effects were exacerbated by the presence of mental health conditions and low socioeconomic status.

**Conclusion:**

Having multiple physical health conditions (physical multimorbidity) creates substantial health and financial burdens on individuals, the health system, and society, including increased use of health services, loss of work productivity, and decreased health-related quality of life. The adverse effects of multimorbidity on health, quality of life, and economic well-being are exacerbated by the co-occurrence of mental health conditions and low socioeconomic status.

**Summary**

**What is already known on this topic?**

Empirical studies suggest that multiple physical health conditions (physical multimorbidity) coupled with a mental health condition are associated with a wide range of adverse health, economic, and social outcomes.

**What is added by this report?**

After adjusting for confounding factors, our study showed that physical multimorbidity accompanied by mental health conditions and low socioeconomic status increased the use of health care services while reducing work productivity and health-related quality of life.

**What are the implications for public health practice?**

The Australian health system should prioritize improving the management of people with multimorbidity by using a more patient-centered approach that fosters integration of treatments for physical and mental health conditions.

## Introduction

Chronic health conditions account for most of the world’s premature deaths and through their combined social, cultural, and economic effects are also major contributors to socioeconomic inequalities. The rise in morbidity and mortality associated with these conditions is influenced by population ageing and by socioeconomic, societal, and lifestyle changes (1), factors that also contribute to the increase in the prevalence of multimorbidity (defined as the presence of 2 or more health conditions) (2). In Australia, approximately half the population has at least 1 health condition, and 90% of deaths are due to them (3).

Multimorbidity is associated with a wide range of adverse health, economic, and social outcomes; people with multimorbidity have more complex health care needs (use multiple health services and treatments), poorer physical functioning, and increased disability and mortality (4,5). Multimorbidity is also associated with increased economic costs (both medical and nonmedical) and out-of-pocket spending for medical care (6–9). The effect of multimorbidity on financial status as a result of treatment costs has been well documented (10,11). Less is known about other social effects of multimorbidity, such as its effect on loss of work productivity.

Multimorbidity can involve combinations of both physical and mental health conditions, which often have complex and bidirectional interrelations (12). These combinations can exacerbate disease burdens and socioeconomic outcomes (13–15).

In our study, we used nationally representative survey data from Australia 1) to examine the distribution of physical multimorbidity in relation to the presence of mental health conditions by socioeconomic status and 2) to measure the economic burden of combined physical multimorbidity and mental health conditions on health-related quality of life (HRQoL), health service use, and loss of work productivity.

## Methods

### Sample and data

**Figure 1:**
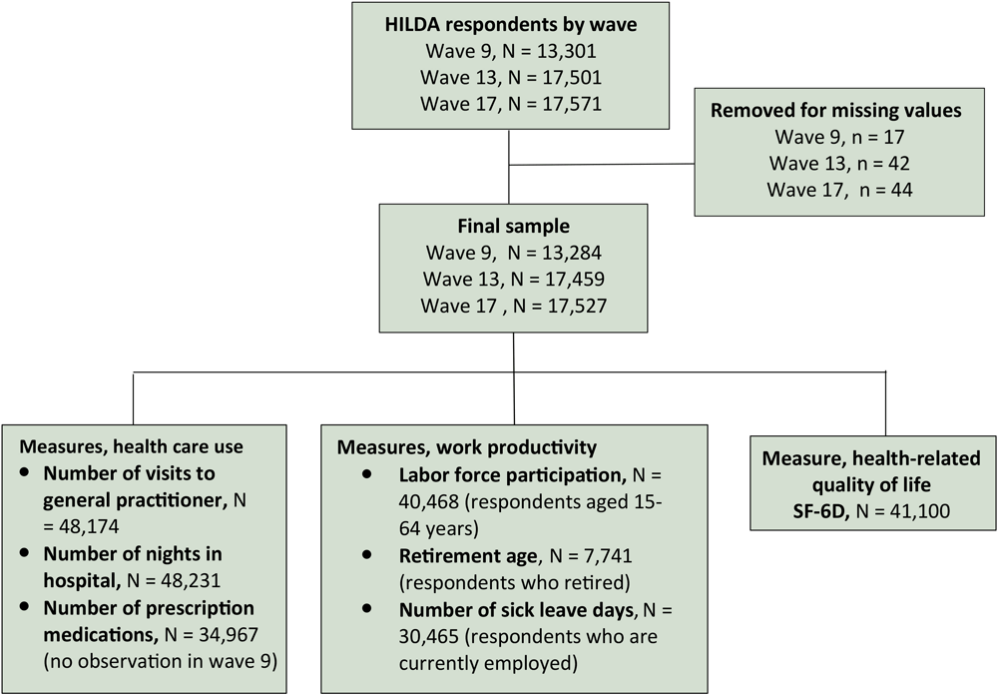
Flowchart showing selection of respondents from HILDA Survey and measures used to evaluate health service use, loss of work productivity, and reduced health-related quality of life (HRQoL). To assess HRQoL we used the SF-6D, which consists of 11 questions in 6 domains (physical functioning, role limitation, social functioning, pain, mental health, and vitality) from the SF-36, the 36-item short form questionnaire for evaluating HRQoL (17).

We used panel data from 3 waves of the Household, Income and Labour Dynamics in Australia (HILDA) Survey conducted in 2009, 2013, and 2017 (16). Briefly, the HILDA Survey is a nationally representative longitudinal survey that collects information on economic and personal well-being, labor market dynamics, and family life in Australian households among individuals aged 15 years or over. Commenced in 2001, the HILDA Survey collects data from the same households and individuals for each year of the survey. A detailed description of survey objectives and methods are provided elsewhere (16). Waves 9, 13, and 17 of the HILDA Survey comprised 13,301, 17,501, and 17,571 respondents, respectively. In our analysis, we included respondents aged 15 or older and excluded those who had missing values on independent variables and health conditions. This left a study sample of 13,284 respondents for wave 9, 17,459 for wave 13, and 17,527 for wave 17 (Figure 1).

### Variables

**Multimorbidity.** We assessed whether respondents had both physical multimorbidity and mental health conditions. The list of physical health conditions included in the HILDA Survey were arthritis/osteoporosis, asthma, cancer, chronic bronchitis/emphysema, type 1 diabetes, type 2 diabetes, heart disease, high blood pressure/hypertension, and any other serious circulatory condition. Mental health conditions included in the HILDA Survey were depression/anxiety and “other mental illnesses.” Respondents who answered affirmatively to the question “Have you been told by a doctor or nurse that you have any of these conditions?” were defined as reporting a health condition. We counted the number of self-reported physical health conditions, excluding mental health conditions, to quantify the number of physical health conditions and categorized people with more than 1 physical health condition as having physical multimorbidity (0 = no physical multimorbidity, 1 = physical multimorbidity). Because the HILDA Survey did not ask about type of mental health condition apart from depression and anxiety, we were not able to create a count variable for mental health conditions. Instead, we included the presence of any mental health condition as a separate binary variable (0 = no mental health condition1 = mental health condition).

**Outcomes.** We examined 3 types of costs associated with physical and mental multimorbidity, including 1) direct costs measured by health service use, 2) indirect costs measured by work productivity loss, and 3) intangible costs measured by health-related quality of life (HRQoL). Use of health services was measured by the number of visits to a general practice (GP) physician in the past 12 months, the number of overnight stays in a hospital in the past 12 months, and the number of reported prescription medications taken on a regular basis in the past 12 months (16). Respondents who reported 5 or more prescription medications were assigned polypharmacy status, and those who reported 10 or more prescription medications were assigned excessive polypharmacy status. Second, work productivity was measured by labor force participation status, retirement age, and mean number of days of sick leave taken per year. Labor force participation status was defined as the respondent’s employment status (in labor force, not in labor force) at the time of the survey. Respondents were categorized in “labor force participation” if they were either currently working or were unemployed but actively looking for a job. Retirement age was defined as the age when the respondent retired. This question was asked only of respondents who were retired at the time of the survey. Absence from work was assessed on the basis of the number of paid sick leave days taken by respondents who were employed at the time of the survey. Finally, to assess HRQoL we used the SF-6D, which consists of 11 questions in 6 domains (physical functioning, role limitation, social functioning, pain, mental health, and vitality) from the SF-36, the 36-item short form questionnaire for evaluating HRQoL (17). The SF-6D provided a nondisease-specific measure of respondents’ health status and experience and assigned a continuous value between 0 (worst health state) and 1 (best health state).

**Covariates.** Control variables included for the analysis were sex, age (age in years at wave 9), education level (low level, year 11 and below; middle level, year 12, certificate iii or ⅳ, diploma, advanced diploma; high level, bachelor or honors, graduate diploma, graduate certificate, post graduate); Indigenous status (non-Aboriginal, Aboriginal, and Torres Strait Islander); country of birth (Australia, other English-speaking countries [United Kingdom, New Zealand, Canada, United States, Ireland, or South Africa]; all others); marital status (married/cohabiting, other [single, separated, divorced, widowed]); Australian state (New South Wales, Victoria, Queensland, South Australia, Western Australia, Tasmania, Northern Territory, Australian Capital Territory); residential area (rural, urban); and wave number (coded as 0 in wave 9, 1 in wave 13, and 2 in wave 17). To create the socioeconomic status (SES) quintile, we used Socio Economic Indexes for Areas (SEIFA), a scale of 1 to 5 in which 1 is the lowest SES group and 5 is the highest. SEIFA is an index based on social and economic census data from 2011 that ranks geographic areas across Australia according to relative socioeconomic advantage and disadvantage (18).

### Statistical analysis

Because HILDA data included repeated measurements of individuals over time, we used generalized estimating equations (GEE) with an unstructured covariance matrix and robust standard errors to estimate the outcome–exposure relationship while accounting for within-person correlation. Different families of GEE were used depending on the outcome of interest. The negative binomial family with a logit link function was used for count-based outcomes (number of GP visits, number of nights in hospital, number of prescription medications, number of days of sick leave), and coefficients were interpreted as the increase in number for each unit increase of the associated variable. The binomial family with a logit link function was used to calculate binary outcomes (polypharmacy, excessive polypharmacy, labor force participation) and odd ratios. We used the Gaussian family with an identity link function for continuous outcomes (retirement age, SF-6D score of HRQoL) with coefficients interpreted as linear changes in the outcome. Each model included all covariates. We also examined the longitudinal association between the presence of a mental health condition (at baseline) and physical multimorbidity (over time), and also the association between physical multimorbidity (at baseline) and a mental health condition (over time). For the longitudinal analysis, we used binomial GEEs with a logit link and included time (measured in years from baseline) and the time–exposure interaction in the model.

Additionally, we conducted 2 sets of sensitivity analyses to test the robustness of our models. First, we re-analyzed each association by using balanced samples to compare the results analyzed by using unbalanced samples. Second, we compared the differential effect of mental health conditions by replacing the model’s exposure variable “mental health condition,” which encompassed all mental health conditions, with either 1) depression/anxiety or 2) other mental illnesses. All analyses were performed using Stata 15 (Stata Corp).

## Results

### Sample characteristics

Women accounted for approximately half of the samples. In all 3 waves, most respondents were aged younger than 50, had a middle education level, and were married/cohabiting. Respondents reporting mental health conditions represented 11.2% of the sample in 2009 and 17.3% in 2017. The proportion of respondents with physical multimorbidity was 13.3% in 2009 and 14.5% in 2017 amongst respondents who did not report mental health conditions and 29.5% in 2009 and 24.2% in 2017 amongst those reporting mental health conditions.

**Figure 2:**
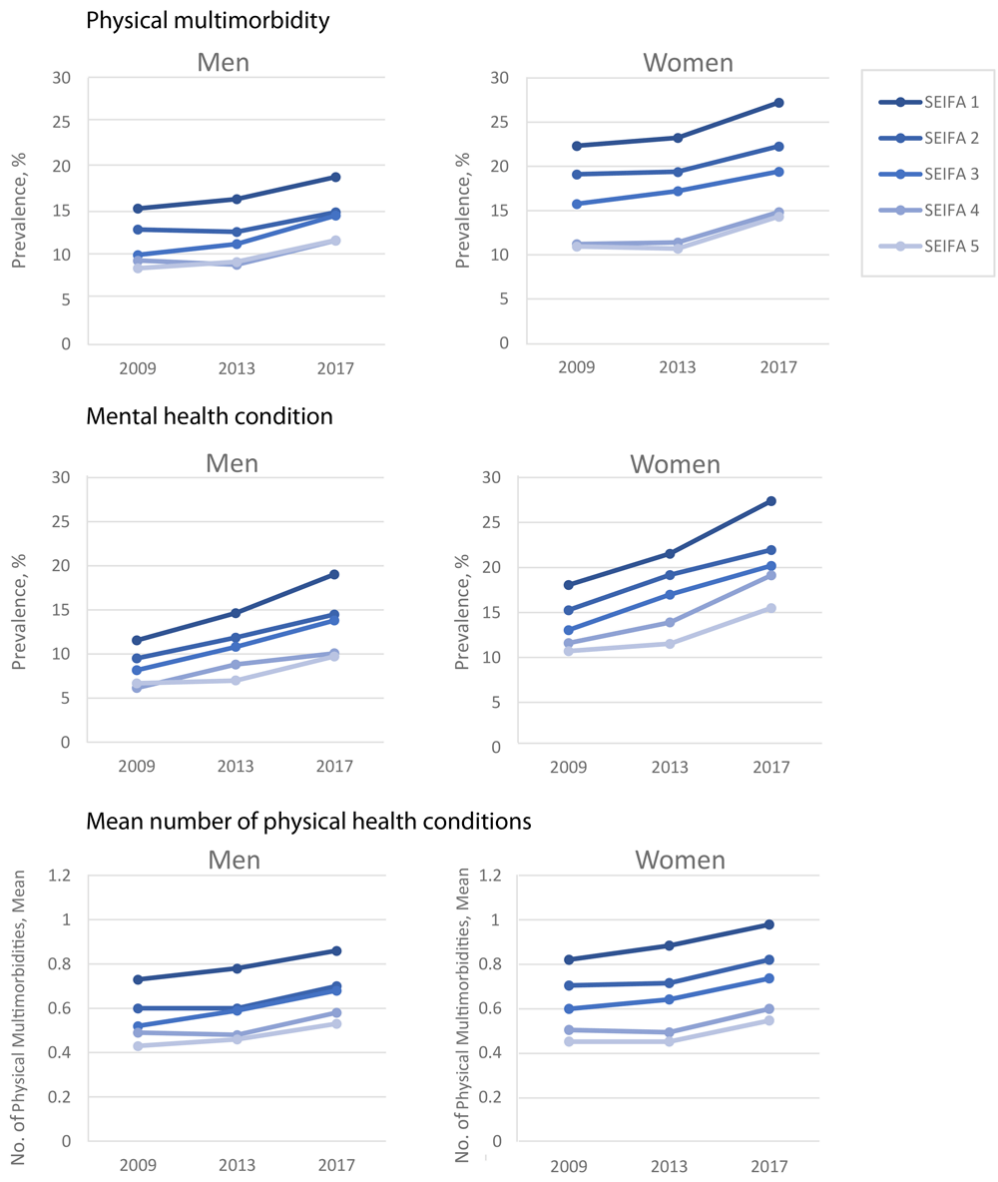
Prevalence of physical multimorbidity and mental health conditions and the mean number of physical health conditions across 3 waves of the Household, Income and Labour Dynamics in Australia (HILDA) Survey, 2009, 2013, and 2017, by sex and socioeconomic status. Socioeconomic status is measured on the SEIFA (Socio Economic Indexes for Areas) scale and ranges from 1 to 5, with 5 being the highest status (18).

**Physical multimorbidity and mental health conditions by SES**. For both men and women, the prevalence of physical multimorbidity was higher among the lowest SES group than among the highest SES group in all waves (Figure 2). For example, 19.1% of male respondents with the lowest SES had physical multimorbidity in 2013 compared with 10.8% among those with the highest SES. We also observed this trend for the prevalence of mental health conditions. The prevalence of mental health conditions among women with the lowest SES (21.5%) was almost double that of respondents with the highest SES (11.5%) in 2013. It is worth noting that the increase in the prevalence of these conditions as well as the number of physical health conditions by waves reflects the ageing effect of the sample.

**Table:** 

HILDA Wave	SEIFA 1	SEIFA 2	SEIFA 3	SEIFA 4	SEIFA 5
Men					
Physical multimorbidity					
9 (2009)	17.8	15.1	11.7	11.0	10.0
13 (2013)	19.1	14.8	13.1	10.5	10.8
17 (2017)	21.9	17.3	16.9	13.6	13.7
Mental health conditions					
9 (2009)	11.54	9.5	8.16	6.14	6.68
13 (2013)	14.6	11.9	10.8	8.8	7.0
17 (2017)	19.0	14.5	13.8	10.1	9.7
Mean number of physical health conditions					
9 (2009)	0.7	0.6	0.5	0.5	0.4
13 (2013)	0.8	0.6	0.6	0.5	0.5
17 (2017)	0.9	0.7	0.7	0.6	0.5
Women					
Physical multimorbidity					
9 (2009)	22.3	19.1	15.8	11.2	11.0
13 (2013)	23.2	19.4	17.2	11.4	10.7
17 (2017)	27.2	22.3	19.4	14.8	14.3
Mental health conditions					
9 (2009)	18.1	15.3	13.1	11.6	10.7
13 (2013)	21.5	19.2	17.0	13.9	11.5
17 (2017)	27.4	22.0	20.2	19.1	15.5
Mean number of physical health conditions					
9 (2009)	0.8	0.7	0.6	0.5	0.5
13 (2013)	0.9	0.7	0.7	0.5	0.5
17 (2017)	1.0	0.8	0.8	0.6	0.6

**Longitudinal association between physical multimorbidity and mental health condition**. We saw a clear association between physical multimorbidity and mental health conditions (Table 1). The presence of a mental health condition was associated with an increased risk of physical multimorbidity (adjusted odds ratio [AOR] = 3.44; 95% CI, 3.00–3.95). Physical multimorbidity was associated with an increased risk of a mental health condition (AOR = 3.10; 95% CI, 2.73–3.53). Adjusting for baseline age, with each year the risk of physical multimorbidity increased with time where there was no mental health condition at baseline (AOR = 1.08; 95% CI, 1.07–1.09). Where there was a mental health condition at baseline, the increased risk over time was negligible (AOR = 1.02; 95% CI, 1.00–1.04). Likewise, the risk of a mental health condition increased with time where there was no physical multimorbidity (AOR = 1.08; 95% CI, 1.07–1.09), and where the risk of physical multimorbidity was smaller (AOR = 1.02; 95% CI, 1.01–1.04).

**Table 1:** Longitudinal Association Between Physical Multimorbidity and Mental Health Condition, HILDA Survey^a^, Australia, 2009, 2013, 2017

Characteristic	Physical Multimorbidity	Mental Health Condition
	AOR (95% CI)	AOR (95% CI)
**Mental health condition (at baseline)**	3.44 (3.00–3.95)	NA
**Physical multimorbidity (at baseline)**	NA	3.10 (2.73–3.53)
**Time (years from baseline)**		
**No mental health condition**	1.08 (1.07–1.09)	NA
**Mental health condition**	1.02 (1.00–1.04)	NA
**No physical multimorbidity**	NA	1.08 (1.07–1.09)
**Physical multimorbidity**	NA	1.02 (1.01–1.04)
**Socioeconomic indexes for areas^b^**		
5	Reference	
4	1.04 (0.93–1.17)	1.1 (0.99–1.21)
3	1.3 (1.16–1.47)	1.25 (1.13–1.38)
2	1.36 (1.21–1.53)	1.34 (1.22–1.48)
1	1.66 (1.47–1.86)	1.54 (1.39–1.69)
**Sex**		
Male	Reference	
Female	1.11 (1.02–1.19)	1.7 (1.59–1.82)
**Baseline age (in years)**	1.09 (1.08–1.09)	0.99 (0.99–0.99)
**Education level^c^**		
Low	Reference	
Middle	0.84 (0.77–0.92)	1.03 (0.96–1.10)
High	0.64 (0.58–0.72)	0.8 (0.73–0.88)
**Indigenous status**		
Non-Indigenous Australian	Reference	
Indigenous Australian	1.73 (1.40–2.14)	1.38 (1.18–1.62)
**Country of birth**		
Australia	Reference	
Other English-speaking country (United Kingdom, New Zealand, Canada, United States, Ireland, or South Africa)	0.93 (0.83–1.05)	1.0 (0.89–1.14)
Other	0.89 (0.80–1.00)	0.68 (0.61–0.77)
**Marital status**		
Married/de facto	Reference	
Single, separated, divorced, widowed	0.99 (0.92–1.07)	1.28 (1.21–1.37)
**State**		
New South Wales	Reference	
Victoria	0.88 (0.80–0.98)	1.13 (1.04–1.24)
Queensland	0.94 (0.85–1.05)	1.02 (0.93–1.11)
South Australia	1.09 (0.95–1.25)	1.14 (1.01–1.29)
Western Australia	0.91 (0.79–1.05)	1.14 (1.01–1.29)
Tasmania	1.16 (0.95–1.42)	1.2 (1.01–1.42)
Northern Territory	0.72 (0.43–1.20)	0.79 (0.55–1.13)
Australian Capital Territory	1.08 (0.80–1.46)	1.17 (0.93–1.47)
**Area**		
Urban	Reference	
Rural	1.01 (0.92–1.11)	0.94 (0.86–1.02)

Abbreviations: AOR, adjusted odds ratio; HILDA, Household, Income and Labour Dynamics in Australia Survey.

a A nationally representative longitudinal survey that collects key information on economic and personal well-being, labor market dynamics, and family life in Australian households among individuals aged 15 years or over (16).

b Scale of 1 to 5 with 5 the highest. Socio Economic Indexes for Areas (SEIFA) is an index that ranks geographic areas across Australia according to relative socioeconomic advantage and disadvantage. SEIFA was created on the basis of 2011 social and economic census information (18).

c Low level (year 11 and below), middle level (year 12, certificate ⅲ or ⅳ, diploma, advanced diploma), high level (bachelor or honors, graduate diploma, graduate certificate, post graduate).

### Association between physical multimorbidity, mental health, and low socioeconomic status

**Health service use**. An increase in the number of physical health conditions was associated with a greater number of GP visits (incidence rate ratio [IRR] = 1.41; 95% CI, 1.39–1.43) and the number of nights in hospital (IRR = 1.77; 95% CI, 1.68–1.87) (Table 2). The presence of a mental health condition was also associated with an increasing number of GP visits (IRR = 2.11; 95% CI, 2.03–2.20) and the number of nights in hospital (IRR = 3.59; 95% CI, 2.93–4.41). Low SES was also associated with the number of GP visits (ie, respondents with the lowest SES had an increased number of GP visits [SEIFA1: IRR = 1.23; 95% CI, 1.19–1.29] compared with those with higher SES.

**Table 2:** Effect of the Association Between Physical Multimorbidity, Mental Health Conditions, and Socioeconomic Status on Health Service Use, HILDA Survey**^a^**, Australia, 2009, 2013, 2017

Characteristic	Number of GP Visits, IRR (95% CI)^b^	Number of Nights at Hospital, IRR (95% CI)^b^	Number of Prescription Medications, IRR (95% CI)^b^	Polypharmacy, AOR (95% CI)^c^	Excessive Polypharmacy, AOR (95% CI)^d^
**Physical multimorbidity**	1.41 (1.39−1.43)	1.77 (1.68−1.87)	1.75 (1.72−1.78)	2.77 (2.63−2.93)	2.48 (2.25−2.73)
**Mental health condition**	2.11 (2.03−2.20)	3.59 (2.93−4.41)	3.08 (2.92−3.25)	4.61 (3.84−5.53)	3.69 (2.46−5.53)
**Physical health conditions × mental health conditions^e^**	0.86 (0.84−0.88)	0.71 (0.65−0.78)	0.73 (0.71−0.75)	0.8 (0.73−0.87)	0.86 (0.76−0.98)
**Survey wave**					
9, 2009, n = 13,284	Reference				
13, 2013, n = 17 459	0.99 (0.97−1.02)	1.05 (0.92−1.19)	Reference		
17, 2017, n = 17,527	0.99 (0.96−1.01)	1.08 (0.95−1.23)	1.06 (1.04−1.09)	1.14 (1.05−1.23)	1.06 (0.89, 1.26)
**Socioeconomic indexes for areas^f^**					
5	Reference				
4	1.05 (1.01−1.09)	0.96 (0.81−1.14)	1 (0.94−1.05)	0.99 (0.82−1.19)	0.89 (0.58−1.37)
3	1.08 (1.04−1.13)	1.27 (1.06−1.52)	1.02 (0.97−1.08)	1.11 (0.92−1.33)	0.9 (0.60, 1.36)
2	1.18 (1.13−1.22)	1.14 (0.96−1.36)	1.11 (1.05−1.17)	1.32 (1.11−1.58)	1.3 (0.88−1.93)
1	1.23 (1.19−1.29)	1.37 (1.13−1.67)	1.12 (1.05−1.18)	1.33 (1.11−1.59)	1.37 (0.93−2.01)
**Sex**					
Male	Reference				
Female	1.41 (1.38−1.45)	1.22 (1.08−1.37)	1.2 (1.16−1.24)	0.83 (0.74−0.92)	0.78 (0.62−0.97)
**Age**	1 (1.00−1.00)	1.02 (1.01−1.02)	1.03 (1.03−1.03)	1.06 (1.05−1.06)	1.04 (1.04−1.05)
**Education^g^**					
Low	Reference				
Middle	1 (0.97−1.03)	0.95 (0.83−1.09)	0.98 (0.94−1.02)	0.84 (0.75−0.95)	0.87 (0.68−1.10)
High	0.88 (0.85−0.91)	0.91 (0.77−1.06)	0.95 (0.90−1.00)	0.66 (0.56−0.78)	0.68 (0.47−1.00)
**Indigenous status**					
Non-Indigenous Australian	Reference				
Indigenous Australian	1.11 (1.02−1.20)	1.67 (1.19−2.34)	1.01 (0.91−1.11)	1.32 (0.97−1.79)	1.03 (0.56−1.90)
**Country of birth**					
Australia	Reference				
Other English-speaking country (United Kingdom, New Zealand, Canada, United States, Ireland, or South Africa)	0.96 (0.91−1.00)	0.82 (0.68−0.98)	0.94 (0.89−0.99)	0.99 (0.84−1.16)	0.99 (0.72−1.36)
All others	1.06 (1.02−1.11)	0.82 (0.65−1.04)	0.82 (0.78−0.86)	0.83 (0.71−0.98)	0.7 (0.50−0.99)
**Marital status**					
Married/cohabiting	Reference				
Single, separated, divorced, or widowed	1.0 (0.97−1.02)	1.24 (1.10−1.39)	1.03 (1.00−1.07)	1.07 (0.96−1.20)	1.34 (1.08−1.66)
**State**					
New South Wales	Reference				
Victoria	1.03 (0.99−1.06)	0.97 (0.82−1.14)	1.01 (0.97−1.06)	1.07 (0.93−1.24)	1.2 (0.89−1.62)
Queensland	1.02 (0.98−1.05)	1.03 (0.88−1.20)	1.03 (0.98−1.08)	1.13 (0.97−1.31)	1.36 (1.01−1.83)
South Australia	1.0 (0.95−1.05)	0.95 (0.78−1.16)	1.08 (1.02−1.15)	1.24 (1.02−1.50)	1.47 (1.02−2.11)
Western Australia	0.95 (0.91−1.00)	0.99 (0.81−1.21)	1.06 (0.99−1.13)	1.18 (0.96−1.44)	1.27 (0.86−1.89)
Tasmania	0.96 (0.89−1.04)	0.77 (0.60−0.99)	1.05 (0.96−1.16)	0.92 (0.67−1.26)	0.85 (0.47−1.53)
Northern Territory	0.88 (0.76−1.01)	1.14 (0.74−1.76)	0.95 (0.73−1.23)	0.71 (0.27−1.90)	NA
Australian Capital Territory	0.96 (0.88−1.05)	1.03 (0.68−1.55)	0.96 (0.84−1.09)	0.97 (0.62−1.51)	1.67 (0.67−4.18)
**Area**					
Urban	Reference				
Rural	0.95 (0.91−0.99)	1.01 (0.86−1.18)	0.97 (0.93−1.02)	0.97 (0.84−1.13)	0.88 (0.64−1.22)

Abbreviations: AOR, adjusted odds ratio; GP, general practitioner; HILDA, Household, Income and Labour Dynamics in Australia Survey; IRR, incidence rate ratio; NA, not applicable.

a A nationally representative longitudinal survey that collects key information on economic and personal well-being, labor market dynamics, and family life in Australian households among individuals aged 15 years or over (16).

b Number of GP visits, nights at hospital, and prescription medications calculated by using generalized estimating equations with negative binomial family.

c Taking 5 or more prescription medications, calculated by using generalized estimating equations with binomial family.

d Taking 10 or more prescription medications, calculated by using generalized estimating equations with binomial family.

e Physical and mental condition interaction: number of physical conditions and the presence of mental health conditions (binary).

f Scale of 1 to 5 with 5 the highest. Socio Economic Indexes for Areas (SEIFA) is an index that ranks geographic areas across Australia according to relative socioeconomic advantage and disadvantage. SEIFA was created on the basis of 2011 social and economic census information.

g Low level (year11 and below), middle level (year 12, certificate ⅲ or ⅳ, diploma, advanced diploma), high level (bachelor or honors, graduate diploma, graduate certificate, post graduate).

An increased number of physical health conditions was associated with an increase in the mean number of prescription medications used (IRR = 1.75; 95% CI, 1.72–1.78), the odds of polypharmacy (AOR = 2.77; 95% CI, 2.63–2.93), and excessive polypharmacy (AOR = 2.48; 95% CI, 2.25–2.73). The presence of mental health conditions was independently associated with the number of prescription medications (IRR = 3.08; 95% CI, 2.92–3.25) and the odds of polypharmacy (AOR = 4.61; 95% CI, 3.84–5.53). Low SES showed an inverse relationship with the number of prescription medications (SEIFA1: IRR = 1.33; 95% CI, 1.11–1.59). We observed significant interactions between physical and mental health conditions, but the absolute difference was small.

**Work productivity**. An increased number of physical health conditions coupled with the presence of mental health conditions and low SES were associated with decreased work productivity (Table 3). For example, the AOR of labor force participation decreased as the number of physical health conditions increased (AOR = 0.71; 95% CI, 0.69–0.74). An increasing number of physical conditions was associated with retirement at a younger age (Coeff = −0.16; 95% CI, −0.25 to −0.07). The mean number of sick leave days taken increased with an increase in the number of physical health conditions (IRR = 1.24; 95% CI, 0.18–1.30). Low SES was also associated with loss of work productivity according to the lower probability of labor force participation and the increasing number of sick leave days taken observed in the lowest SES group. Reporting a mental health condition had no significant association with retirement age but was associated with a low participation in the labor force (AOR = 0.62; 95% CI, 0.57–0.68) and an increasing number of sick leave days taken (IRR = 1.45; 95% CI, 1.27–1.64).

**Table 3:** Effect of the Association Between Physical Multimorbidity, Mental Health Conditions, and Socioeconomic Status on Work Productivity, HILDA Survey**^a^**, Australia, 2009, 2013, 2017

Characteristic	Labor Force Participation, AOR (95% CI)^b^	Retirement Age, Coeff (95% CI)^c^	Number of Sick Leave Days Taken, IRR (95%CI)^d^
**Physical multimorbidity**	0.71 (0.69 to 0.74)	−0.16 (−0.25 to 0.07)	1.24 (1.18 to 1.30)
**Mental health condition**	0.62 (0.57 to 0.68)	−0.35 (−0.85 to 0.14)	1.45 (1.27 to 1.64)
**Physical health conditions × mental health conditions^e^**	0.93 (0.87 to 0.99)	0.05 (−0.20 to 0.30)	0.86 (0.75 to 0.98)
**Survey wave no.**			
9, 2009, n = 13, 284	Reference		
13, 2013, n = 17, 459	0.85 (0.80 to 0.89)	0.87 (0.55 to 1.20)	0.96 (0.90 to 1.02)
17, 2017, n = 17,527	0.88 (0.83 to 0.93)	1.2 (0.86 to 1.54)	1 (0.94 to 1.07)
**Socioeconomic indexes for areas^f^**			
5	Reference		
4	1.22 (1.11 to 1.34)	−0.03 (−0.55 to 0.49)	1.05 (0.96 to 1.14)
3	1.07 (0.98 to 1.18)	0.02 (−0.49 to 0.53)	1.07 (0.98 to 1.16)
2	0.94 (0.86 to 1.03)	−0.33 (−0.84 to 0.17)	1.13 (1.04 to 1.23)
1	0.76 (0.69 to 0.83)	−0.1 (−0.61 to 0.41)	1.17 (1.07 to 1.28)
**Sex**			
Male	Reference		
Female	0.53 (0.49 to 0.56)	−4.77 (−5.43 to −4.12)	1.17 (1.11 to 1.23)
**Age**	0.99 (0.98 to 0.99)	0.1 (0.07 to 0.14)	1.01 (1.00 to 1.01)
**Education^g^**			
Low education	Reference		
Middle education	2.65 (2.48 to 2.84)	2.83 (2.09 to 3.57)	1.42 (1.31 to 1.53)
High education	4.23 (3.85 to 4.65)	5.72 (4.85 to 6.59)	1.61 (1.47 to 1.77)
**Indigenous status**			
Non-Indigenous Australian	Reference		
Indigenous Australian	0.57 (0.49 to 0.66)	−1.28 (−4.85 to 2.29)	1.24 (1.07 to 1.44)
**Country of birth**			
Australia	Reference		
Other English-speaking country (United Kingdom, New Zealand, Canada, United States, Ireland, or South Africa)	0.97 (0.86 to 1.09)	1.07 (0.23 to 1.90)	0.94 (0.83 to 1.07)
All others	0.59 (0.53 to 0.65)	−0.36 (−1.24 to 0.51)	0.83 (0.76 to 0.91)
**Marital status**			
Married/living together	Reference		
Single, separated, divorced, or widowed	0.71 (0.67 to 0.75)	−0.33 (−0.71 to 0.05)	0.8 (0.76 to 0.86)
**State**			
New South Wales	Reference		
Victoria	1.08 (1.00 to 1.17)	0.27 (−0.50 to 1.03)	1.04 (0.97 to 1.13)
Queensland	1.13 (1.04 to 1.23)	−0.08 (−0.81 to 0.66)	0.98 (0.91 to 1.06)
South Australia	1.07 (0.95 to 1.19)	−0.17 (−1.21 to 0.87)	1.03 (0.91 to 1.17)
Western Australia	1.12 (0.99 to 1.26)	0.93 (−0.13 to 1.99)	0.99 (0.89 to 1.11)
Tasmania	1.28 (1.07 to 1.52)	−0.03 (−1.47 to 1.41)	1.03 (0.88 to 1.21)
Northern Territory	2.74 (1.67 to 4.47)	−4.2 (−13.99 to 5.58)	1.44 (1.13 to 1.84)
Australian Capital Territory	1.23 (0.96 to 1.57)	NA	1.51 (1.30 to 1.76)
**Area**			
Urban	Reference		
Rural	0.87 (0.80 to 0.95)	0.54 (0.03 to 1.06)	0.8 (0.71 to 0.90)

Abbreviations: AOR, adjusted odds ratio; HILDA, Household, Income and Labour Dynamics in Australia Survey; IRR, incidence rate ratio; NA, not applicable.

a A nationally representative longitudinal survey that collects key information on economic and personal well-being, labor market dynamics, and family life in Australian households among individuals aged 15 years or over (16).

b Respondents aged ≤65 who participated in the labor force, calculated by using generalized estimating equations with binomial family.

c Respondents aged ≥65 who were retired from the labor force, calculated by using generalized estimating equations with Gaussian family.

d Calculated by using generalized estimating equations with Gaussian family.

e Physical and mental condition interaction: number of physical conditions and the presence of mental health conditions (binary).

f Scale of 1 to 5 with 5 the highest. Socio Economic Indexes for Areas (SEIFA)is an index that ranks geographic areas across Australia according to relative socioeconomic advantage and disadvantage. SEIFA is based on 2011 social and economic census information (18).

g Low level (year 11 and below), middle level (year 12, certificate ⅲ or ⅳ, diploma, advanced diploma), high level (bachelor or honors, graduate diploma, graduate certificate, post graduate).

**Health-related quality of life.** For each increase in the number of physical health conditions, a substantial reduction in SF-6D scores (Coeff = −0.03; 95% CI, −0.03 to −0.03 of the total scale of 1) occurred (Table 4). Regardless of the number of physical health conditions, reporting a mental health condition decreased HRQoL (Coeff = −0.1; 95% CI, −0.1 to −0.09). The association between SES and SF-6D score showed that the low SES group had low HRQoL (SEIFA1: Coeff = −0.02; 95% CI, −0.03 to −0.02).

**Table 4:** Effects of Association Between Physical Multimorbidity, Mental Health Conditions, and Socioeconomic Status on Health-Related Quality of Life, HILDA Survey^a^, Australia, 2009, 2013, 2017

Characteristic	SF-6D^b^
	Coefficient (95% CI)^c^
**Physical multimorbidity**	−0.03 (−0.03 to −0.03)
**Mental health condition**	−0.1 (−0.10 to−0.09)
**Physical health conditions × mental health conditions^d^**	0 (0.00 to 0.01)
**Survey wave no.**	
9, 2009, N = 13,284	Reference
13, 2013, N = 17,459	−0.01 (−0.01 to −0.00)
17, 2017, N = 17,527	−0.01 (−0.01 to −0.01)
**Socioeconomic indexes for areas^e^**	
5	Reference
4	−0.01 (−0.01 to −0.00)
3	−0.01 (−0.01 to −0.01)
2	−0.02 (−0.02 to −0.01)
1	−0.02 (−0.03 to −0.02)
**Sex**	
Male	Reference
Female	−0.01 (−0.02 to −0.01)
**Age**	0 (−0.00 to −0.00)
**Education^f^**	
Low education	Reference
Middle education	0.01 ((0.00 to 0.01)
High education	0.02 ((0.01 to 0.02)
**Indigenous status**	
Non-Indigenous Australian	Reference
Indigenous Australian	−0.02 (−0.03 to −0.01)
**Country of birth**	
Australia	Reference
Other English-speaking countries (United Kingdom, New Zealand, Canada, United States, Ireland, or South Africa)	0 (−0.00 to 0.01)
All others	−0.02 (−0.02 to −0.01)
**Marital status**	
Married/cohabiting	Reference
Single, separated, divorced, or widowed	−0.01 (−0.02 to −0.01)
**State**	
New South Wales	Reference
Victoria	0 (−0.00 to 0.00)
Queensland	0 (−0.01 to −0.00)
South Australia	−0.01 (−0.01 to −0.00)
Western Australia	0 (−0.01 to 0.00)
Tasmania	0.01 (−0.00 to 0.01)
Northern Territory	0 (−0.02 to 0.01)
Australian Capital Territory	−0.01 (−0.01 to 0.00)
**Area**	
Urban	Reference
Rural	0 (−0.00 to 0.00)

Abbreviation: HILDA, Household, Income and Labour Dynamics in Australia Survey.

a A nationally representative longitudinal survey that collects key information on economic and personal well-being, labor market dynamics, and family life in Australian households among individuals aged 15 years or over (16).

b SF-6D is 11 questions from the SF-36 (the short form of the Health Status Questionnaire) used to define the 6 domains of health-related quality of life (physical functioning, role limitation, social functioning, pain, mental health, and vitality) (17). Calculated by using generalized estimating equations with Gaussian family.

c Coefficient was calculated from multivariable linear regression model.

d Physical and mental condition interaction: number of physical condition and the presence of mental health conditions (binary).

e Scale of 1 to 5 with 5 the highest. Socio Economic Indexes for Areas (SEIFA) is an index that ranks geographic areas across Australia according to relative socioeconomic advantage and disadvantage. SEIFA is based on 2011 social and economic census information (18).

f Low level (year 11 and below), middle level (year 12, certificate ⅲ or ⅳ, diploma, advanced diploma), high level (bachelor or honors, graduate diploma, graduate certificate, post graduate).

**Sensitivity analyses.** Changing mental health condition to “depression/anxiety” did not substantially change the results; however, when the same exposure was changed to “other mental illnesses,” the estimated coefficient was slightly larger in the models for the number of nights in hospital (IRR = 10.7; 95% CI, 7.27–15.7), polypharmacy (AOR = 6.95; 95% CI, 4.89–9.86), and labor force participation (AOR = 0.34; 95% CI, 0.28–0.41). In general, the results did not substantially change when we used balanced samples instead of unbalanced samples.

## Discussion

Ours was the first study to use nationally representative data from Australia to examine how physical multimorbidity coupled with a mental health condition is associated with use of health services, work productivity, and HRQoL in relation to low socioeconomic status. Physical multimorbidity and mental health condition were shown to be positively associated at baseline, and also over time (adjusting for age). The reasons why the risk of developing physical multimorbidity is higher over time in the absence of a mental health condition at baseline (and vice versa) may be because people with either physical multimorbidity or a mental health condition are more engaged with health services than those without these conditions. However, we could not confirm this by the modelling approach we used.

We found that physical multimorbidity was associated with increased use of health services and prescription medications, reduced work productivity, and reduced HRQoL. Our study showed that the presence of mental health conditions and low socioeconomic status exacerbated these effects after adjusting for covariates. Collectively, our results suggest that people with the most physical health conditions and mental health conditions in the lowest SES group used the most health services, had the lowest work productivity, and had the lowest HRQoL.

Our study had limitations. The data we collected on physical and mental health conditions were based on self-reported medical history, which may not accurately reflect health status and was likely under-reported, particularly by people from low socioeconomic backgrounds (19,20). Self-reporting of health service use and sick leave days taken in the past 12 months was prone to recall error. The GP visits, overnight stays in hospital, and work productivity loss resulting from sickness that we assessed in our study were not specific to chronic physical health conditions or mental health conditions alone and could have included acute conditions. Patients with severe illness may have been less likely to participate in the survey; therefore, the prevalence and outcomes reported in our study might be underestimated. We used a simple count of physical health conditions to determine multimorbidity and a dichotomous variable for the presence of a mental health condition; therefore, we were not able to account for disease severity. In addition, respondents were asked if they had “depression or anxiety” or “other mental illness” in the HILDA Survey; therefore, we were not able to analyze specific mental health conditions. Furthermore, we used SEIFA as an indicator of SES, which is an index for geographic areas, and might not be a true estimation of individual SES. Finally, we assessed the trajectory of a combined physical multimorbidity and a mental health condition over time, which accounted for a relevant health condition from baseline data (wave 9) but did not account for time-varying exposure after that. Also, our analysis excluded observations with missing data and only accounted for those with complete data.

Our study provides the first comprehensive analysis to consider all types of costs (ie, direct, indirect, and intangible costs) associated with physical and mental multimorbidity by using a large sample nationally representative of Australia. To our knowledge, only 3 studies — from Canada (21), Scotland (22), and France (23) — assessed the joint effect of physical multimorbidity and mental health conditions. Despite differences in the methodologies, our findings were consistent with those results in previous studies indicating that the presence of a mental health condition increased the association between physical multimorbidity and health service use and HRQoL.

Our study contributes to the growing evidence base that multimorbidity is associated with a greater social and financial burden on individuals, especially when they also have a mental health condition. Our findings are consistent with those of previous local studies in Australia and Europe that concluded that multimorbidity places a substantial burden on health service use (24–26). Current health care is based on single-disease–specific care rather than patient-centered care, which takes into account multimorbidity. As a result, clinical care becomes more complex for patients with multiple diseases, as our finding of greater use of health services and polypharmacy for people with multimorbidity illustrates. Therefore, updating clinical guidelines to reflect patient-centered care and multimorbidity, rather than the current single-disease focus, is warranted (27). Very little evidence exists in the literature on the impact of multimorbidity on loss of work productivity. Consistent with our findings, a US study based on a sample from employed nonelderly adults found that multimorbidity was associated with loss of work productivity (28). Our findings on work productivity indicate employees presenting with multimorbidity may have reduced employment prospects, because they are likely to experience difficulties in staying at work or returning to work while using health services and maintaining their health conditions. The cumulative effect of multimorbidity poses further financial burdens on patients with multimorbidity, particularly for those with low SES, who are more likely to have both physical and mental multimorbidity. Furthermore, our study on HRQoL is consistent with a Southern Australian study that found multimorbidity was associated with a lower HRQoL (29).

Our study provides further evidence that suggests targeted policies and interventions should be considered to tackle the growing burden of physical and mental multimorbidity. Despite the growing prevalence of physical and mental multimorbidity, most clinical practice and preventive strategies throughout the world to date emphasize improving identification and management of a single chronic condition (30). Our findings suggest more focus should be placed on treating patients with multimorbidity with a more patient-centered approach that fosters integrated treatment of physical and mental health conditions. It is worth noting that in Australia, patients living in low SES areas tend to receive poorer quality of care than those living in more affluent areas (31). Decisive action is needed to improve the management of chronic conditions for people in low SES areas in order to mitigate socioeconomic inequalities in health and health care.

Our findings support the earlier finding of a negative association between work productivity and multimorbidity (28). Work management plans for employees with multimorbidity that allow flexible work time and workplace adaptation should be considered. Such plans will ensure that patients can get treatment while working and can return to work after long-term leave. Our findings of increased health service costs and lowered work productivity raise the need for health financing policies to alleviate financial burdens amongst people with multimorbidity. Initiatives such as reduced cost-sharing could also be considered. In Australia, this could be achieved by extending the criteria for receiving Australia’s Health Care Card that provides access to reduced costs on medicines, or by considering increasing rebates via the personal income tax. Future research is needed to examine in more detail the impoverishing effect of physical and mental multimorbidity in order to develop suitable policies that protect the health and socioeconomic well-being of people with multimorbidity.
